# Supplementary material for: A Global Screen for Assembly State Changes of the Mitotic Proteome by SEC-SWATH-MS
Source: Cell Syst. 2020 Feb 26;10(2):133–155.e6. doi: 10.1016/j.cels.2020.01.001 (PMC7042714; doi:10.1016/j.cels.2020.01.001)

P98175 | RBM10_HUMAN | RBM10 DXS8237E GPATC9 GPATCH9 KIAA0122
Monomer MW [kDa]: 103.533 Monomer expected elution fraction: 40

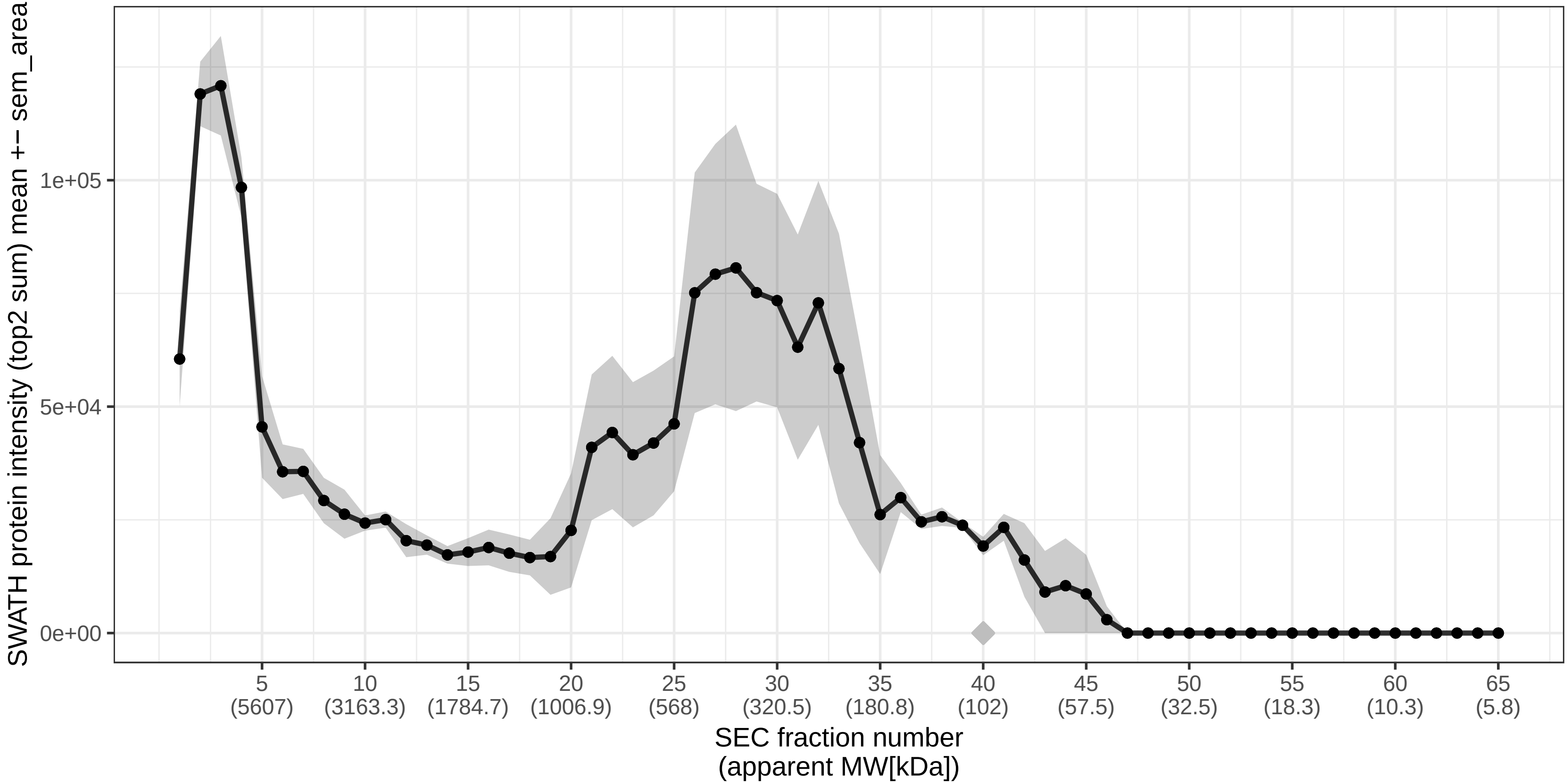

Supplement: Data S1. SEC-SWATH-MS Protein Chromatograms, Related to Figure 1 [file mmc6.zip › SECchrom_P98175_RBM10_HUMAN_RBM10_DXS8237E_GPATC9_GPATCH9_K.pdf]
